# A Compendium of *Caenorhabditis elegans* RNA Binding Proteins Predicts Extensive Regulation at Multiple Levels

**DOI:** 10.1534/g3.112.004390

**Published:** 2013-02-01

**Authors:** Alex M. Tamburino, Sean P. Ryder, Albertha J. M. Walhout

**Affiliations:** * Program in Systems Biology and Program in Molecular MedicineUniversity of Massachusetts Medical School, Worcester, Massachusetts 01605; † Department of Biochemistry and Molecular Pharmacology, University of Massachusetts Medical School, Worcester, Massachusetts 01605

**Keywords:** RNA binding protein, gene expression, regulation, systems biology, *C. elegans*, RBP

## Abstract

Gene expression is regulated at multiple levels, including transcription and translation, as well as mRNA and protein stability. Although systems-level functions of transcription factors and microRNAs are rapidly being characterized, few studies have focused on the posttranscriptional gene regulation by RNA binding proteins (RBPs). RBPs are important to many aspects of gene regulation. Thus, it is essential to know which genes encode RBPs, which RBPs regulate which gene(s), and how RBP genes are themselves regulated. Here we provide a comprehensive compendium of RBPs from the nematode *Caenorhabditis elegans* (wRBP1.0). We predict that as many as 887 (4.4%) of *C. elegans* genes may encode RBPs ~250 of which likely function in a gene-specific manner. In addition, we find that RBPs, and most notably gene-specific RBPs, are themselves enriched for binding and modification by regulatory proteins, indicating the potential for extensive regulation of RBPs at many different levels. wRBP1.0 will provide a significant contribution toward the comprehensive delineation of posttranscriptional regulatory networks and will provide a resource for further studies regulation by RBPs.

Generating the right protein at the right place, the right time, and the right levels is critical during all aspects of life. Multiple levels of gene regulation coordinate the precise expression of genes throughout development and in response to environmental cues and insults. In genomics and systems biology, much attention has focused on the elucidation of regulatory networks involving transcription factors (TFs) or microRNAs (miRNAs) (Martinez and Walhout 2009; Arda and Walhout 2010). These networks include interactions in which these factors both regulate and are regulated by other molecules (Reece-Hoyes *et al.* 2011; Bartel 2009; Deplancke *et al.* 2006; Martinez *et al.* 2008; Harbison *et al.* 2004; Arda *et al.* 2010). RNA binding proteins (RBPs) are another important class of gene regulators; however, the regulatory networks in which they function remain largely uncharacterized.

Although TFs bind DNA and miRNAs interact with mRNAs, RBPs can interact with the entire spectrum of RNAs. These RNAs occur throughout the cell and can take on a vast array of functions, including serving as templates for protein synthesis (mRNA), participating as structural components of the splicing and translation machinery (rRNA, tRNA, snRNA), and providing regulatory activity to modulate transcription, translation and chromatin structure (miRNA, siRNA, piRNA, lncRNA) (Lee and Schedl, 2005 Steitz 2008; Moore and Proudfoot 2009; Carthew and Sontheimer 2009; Wahl *et al.* 2009). Physical interactions between RNA and RBPs are crucial to RNA regulation, for instance, to mediate precise mRNA 3′ end formation, splicing, localization, stability, and translation. As a result of these physical interactions, RBPs can control transcript localization, levels, and translation (Shepard *et al.* 2003; Glisovic *et al.* 2008).

In contrast to RBPs, TFs are rapidly being characterized at a systems level using genome-scale methods such as chromatin immunoprecipitation (ChIP) and yeast one-hybrid assays (Walhout 2011). Among other findings these studies have demonstrated degenerate DNA binding of TFs, extensive combinatorial complexity of interactions between TFs and gene promoters, as well as both specific and promiscuous protein interactions between divergent members of the same TF family (Deplancke *et al.* 2006; Badis *et al.* 2009; Grove *et al.* 2009; Zinzen *et al.* 2009). The systems-level characterization of TFs has been greatly facilitated by high-confidence predictions of which genes in a genome encode such proteins (Reece-Hoyes *et al.* 2005; Kummerfeld and Teichmann 2006; Vaquerizas *et al.* 2009). However, such compendia are not yet available for RBPs in multicellular model organisms.

Here, we present a compendium of predicted RBPs for the nematode *Caenorhabditis elegans* (wRBP1.0). We have used wRBP1.0 to begin the analysis of RBPs at a genome-wide level, using publicly available datasets. We found that RBP-encoding mRNAs have more alternative isoforms, longer 3′ untranslated regions (UTRs), and more alternative polyadenylation (APA) sites than other mRNAs. In addition, RBP gene promoters interact with more TFs, RBP mRNAs are bound by more RBPs, and the 3′ UTRs of RBP-encoding mRNAs are targeted by more miRNAs. Finally, RBPs are phosphorylated more frequently than other proteins. Together, our compendium and analyses provide a first step toward the characterization of RBP regulatory networks in *C. elegans* and serve as a model for the continued study of RBPs in other organisms, including humans.

## Materials and Methods

wRBP1.0 was curated by computationally predicting RNA binding domain (RBD)-containing proteins in the *C. elegans* proteome (WS219). A FASTA file containing the amino acid sequences of all protein coding isoforms in the WS219 release was downloaded from WormBase (http://wormbase.org/). This file was analyzed using a locally installed Unix version of the InterProScan software [iprscan v4.6; InterPro release 24.0; accessed September, 15, 2010 (Quevillon *et al.* 2005; Hunter *et al.* 2009)] using default settings. Iprscan takes the amino acid sequence of each protein as its input and then uses several different applications to search specific databases of domain signatures. The output of iprscan is all recognizable protein domains in that protein sequence. The results were filtered to include only those domains that were identified by Pfam, SMART, Superfamily, or ProSite (Wilson *et al.* 2009; Sigrist *et al.* 2010; Letunic *et al.* 2012; Punta *et al.* 2012) because these applications were most effective at detecting RBDs (data not shown). Results were then manually filtered to include only those proteins that possess one or more of 17 RBDs (Supporting Information, Table S1). Of note is the RGG box, an RBD that was not included as an independent entry by any of the tools used (although it is contained within the specific Pfam domain definition FXR1P_C which encompasses two RRM domains and one RGG box). Although these domains are known RBDs (Kiledjian and Dreyfuss 1992), their sequence and structural determinants have not been well defined. We therefore only included RGG box proteins in our list that have been implicated in the literature as RNA binding. The list was manually checked to verify the presence of known *C. elegans* RBPs and to eliminate false-positive predictions, as enumerated to follow. Five proteins were removed from wRBP1.0 because the computationally predicted domains were much longer or shorter than known RBDs, and we were not confident in their predictions as RBDs based upon visual assessment (R12B2.5, T03G11.3, D2005.1, Y82E9BR.19, and R11H6.5). One protein was removed because it is currently annotated as a pseudogene in Wormbase (C06A1.4). Two proteins were removed due to the lack of characteristic zinc finger homology (Y60A9.3, R03D7.7). We added 12 RBPs based upon published reports that demonstrated or strongly predicted RNA binding (C18G1.4, C50E10.4, M04B2.1, R06F6.1, R144.7, T12F5.5, Y18D10A.17, Y48G8AL.6, Y53C12B.3, ZK1127.1, ZK1236.3, ZK381.4). Sixteen RBPs were added after secondary searches of genes annotated as ‘RNA-binding’ according to Gene Ontology, UniProtKB, or Wang *et al.* 2009 (Gene Ontology Consortium 2000; Uniprot Consortium 2009; Wang *et al.* 2009) based upon manual inspection of all 96 RBPs using information found on Wormbase.org. Wormbase indentified several proteins with noncanonical domains including cytidine deaminases (C47D2.2, F49E8.4), translation initation factors (T01C3.7, F53A2.6, R04A9.4, C05D9.5, Y57A10A.30), tRNA binding proteins (C41G7.1, F29C4.6, C49H3.10), and additional general factors (C12D8.11, C41G7.1, F29C4.6, C49H3.10, C11D2.7, C15C6.4, C48B6.2, F08B4.7) that were missed in our initial screen.

Genome-wide datasets were downloaded from their respective databases or publications. TF binding data were obtained from (Gerstein *et al.* 2010). RIP-Chip data for three RBPs were obtained from (Kershner and Kimble 2010; Kim *et al.* 2010; Wright *et al.* 2010). 3′ UTRs were from 3′UTRome annotations, kindly provided by Marco Mangone. These annotations are reflective of two independent large scale datasets (Mangone *et al.* 2010; Jan *et al.* 2011). TargetScan miRNA target predictions were downloaded from http://www.targetscan.org/worm_52/ based on predictions that corresponded to 3′ UTRs determined using 3P-Seq (Jan *et al.* 2011). mirWIP target predictions (Hammell *et al.* 2008) were kindly provided by Molly Hammell. ALG-1 targets were downloaded from the UCSC genome browser using intersection of the ALG-1 binding sites (Zisoulis *et al.* 2010) with a custom track composed of the aforementioned 3′UTRome annotations. Protein phosphorylation sites from synchronized adult worms were obtained from (Zielinska *et al.* 2009). All data were compiled into a local database (Table S3). The number of alternative isoforms was defined as the number of distinct proteins encoded by a single gene according to WormBase annotations. TF and RBP binding events as determined by ChIP-Seq and RIP-Chip were assigned to their respective genes according to the original publications. The number of miRNAs predicted to target each gene was defined as the number of unique miRNA families with one or more conserved sites predicted in any of the gene’s 3′ UTRs. miRNA targeting was defined to affect a gene when 1+ miRNA target site was gained/lost in an alternative 3′ UTR. miRNA predictions are based on data from Jan *et al.* 2011 and therefore the analyses of alternative 3′ UTRs and their effects on miRNA targeting were based upon the same dataset. The number of posttranslational modifications per protein was calculated from the number of unique residues that were phosphorylated. The number of binding events or posttranslational modifications was calculated for each RNA/protein isoform and then combined nonredundantly for each gene. Hypergeometric and Komolgorov-Smirnov tests were performed using R project software (R Core Team 2012).

## Results and Discussion

### wRBP1.0

**Figure 1:**
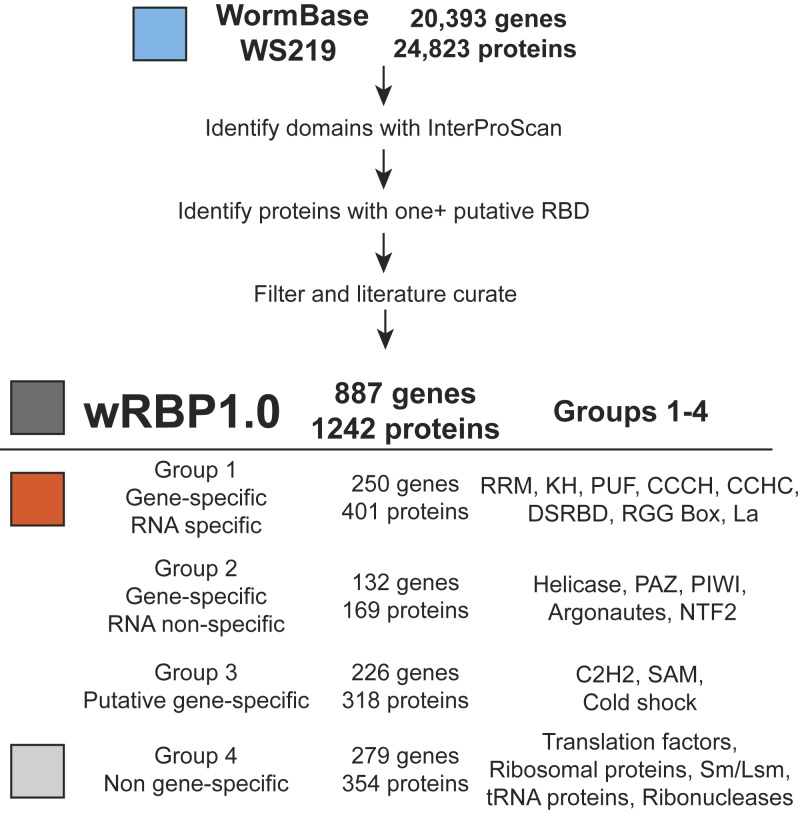
wRBP1.0. Pipeline for *C. elegans* RBP predictions. RBDs were predicted from WormBase protein annotations then filtered and literature curated. RBPs were separated into four groups according to their RBDs as indicated.

To curate the compendium of putative RBPs in *C. elegans*, we searched the proteome (version WS219) for each of 17 RBDs [see the section *RNA binding domains (RBDs)*] based on domain sequence signatures from the unified InterPro database (Quevillon *et al.* 2005; Hunter *et al.* 2009). Proteins were annotated for the presence of each domain using four separate databases (see *Materials and Methods*) and each protein possessing one or more RBD was included in the compendium. Low-confidence calls were removed (see *Materials and Methods*), and the curations were supplemented with RBPs that we identified from the literature but that were missed in the computational search. Of the total RBP set, 67% were identified by more than one method, which illustrates the robustness of our predictions (Figure S1A). Furthermore, the initial list contains greater than 93% of proteins that were previously curated as RNA binding (Wang *et al.* 2009), which illustrates the sensitivity of our method. It is important to note that we increased the number of putative *C. elegans* RBPs by almost threefold relative to this study (from 319 to 887). Two major reasons for this include the inclusion of additional RBDs and protein classes (*i.e.*, dsRBDs, ribosomal proteins, C2H2 zinc fingers, SAM domains) and the inclusion of additional RBPs possessing each domain (*i.e.*, 10–60% increase in KH, RRM, helicase, and CCCH zinc finger domain containing proteins). Further, 66% of the RBPs (177 of 269) annotated in Gene Ontology and UniProtKB databases as ‘RNA binding’ were included, again demonstrating high sensitivity (Figure S1B) (Gene Ontology Consortium 2000; Uniprot Consortium 2009). Next, we manually evaluated 96 RBPs that were not included in our initial list but that were annotated as RNA binding by Gene Ontology, UniProtKB and Wang *et al.* 2009. After careful consideration, we judged 16 of these to be candidate RBPs, whereas we did not have sufficient confidence to include the other 80 (data not shown). Finally, we determined that wRBP1.0 includes 220 of 230 protein listed in RBPDB (Cook *et al.* 2011) including 22 of 23 proteins with experimental evidence of RNA binding [AIN-1 is associated with the miRNA silencing complex but does not require RNA for binding (Wormbase.org)]. Altogether, this generated a final wRBP1.0 compendium of 887 genes. RBPs were then classified into Groups 1-4 based on the domains they possess (Figure 1, see below).

### RNA binding domains (RBDs)

We identified a set of 17 RBDs by literature searches for proteins that bind to RNA (Figure 1, Table S1). Altogether, we identified 887 putative RBP-encoding genes (Table S2; see below). We divided these genes into four groups based on whether they are more likely to bind and regulate RNA in a gene-specific or nonspecific manner. Many RBPs contain multiple RBDs; however, only 10 of 887 genes contain domains from two or more different groups (Table S2). The classification of these 10 genes was first based on the presence of a sequence- or structure-specific RBD.

#### Group 1: Gene-specific RBDs that bind RNA in a sequence-specific manner:

This group contains eight RBDs that mediate binding to specific mRNAs in a sequence and/or structure-specific manner (Figure 1). RNA binding by these domains has been demonstrated for several individual proteins *in vitro*, and gene-specific binding has been detected for several proteins *in vivo* (Table S1) (Ryder *et al.* 2004; Bernstein *et al.* 2005; Opperman *et al.* 2005; Pagano *et al.* 2007; Farley *et al.* 2008; Pagano *et al.* 2009; Kershner and Kimble 2010; Wright *et al.* 2010). Direct, sequence-specific RNA binding has been shown for some *C. elegans* RBPs, but the vast majority remains untested. For instance, GLD-1 (KH domain) and FBF-1 both bind specific sequences *in vitro* (Ryder *et al.* 2004; Bernstein *et al.* 2005) and associate with specific mRNAs *in vivo* (Kershner and Kimble 2010; Wright *et al.* 2010; Jungkamp *et al.* 2011). Altogether, 250 of the 887 RBP-encoding genes are included in Group 1.

#### Group 2: Gene-specific RBDs that do not bind RNA in a sequence-specific manner:

RBDs within Group 2 bind RNA in a gene-specific manner *in vivo*. However, contrary to Group 1 RBDs, the means for this RNA binding specificity are unknown or occur in a manner that is not inherent to the RBD itself (*i.e.*, the domain contributes to RNA binding affinity rather than specificity). For instance, the argonautes ALG-1 and ALG-2 bind miRNAs through their PAZ/PIWI domains. Complementary base pairing by these miRNAs directs targeting of these proteins to specific mRNAs. Out of the 17 RBDs considered, four are placed in this group: helicase, PAZ, PIWI, and NTF2, altogether encoding 169 proteins.

#### Group 3: Putative gene-specific RBDs:

Group 3 proteins are predicted to bind RNA in a gene- and sequence-specific manner. However, we have separated Group 3 proteins from those in Group 1 because their RBDs could be involved not only in RNA binding but also in DNA binding, or protein-protein interactions, thus making the prediction of their function ambiguous (see Table S1 for references). For instance, *Xenopus laevis* TFIIIA can bind both DNA and RNA through various combinations of its C2H2 zinc fingers (Theunissen *et al.* 1992; Lu *et al.* 2003). All proteins with the domains of group 3 are included although we expect that not all of them will mediate RNA binding (*e.g.*, many C2H2 zinc fingers occur in TFs that bind DNA). Group 3 contains three of the 17 RBDs and 226 genes.

#### Group 4: Nongene-specific RBPs, with some exceptions:

The fourth group contains RBDs that typically do not bind RNA in a gene-specific manner. Many essential factors involved in general gene expression are in this group, including ribosomal proteins, transfer RNA-binding proteins, translation initiation factors, core splicing proteins and RNA degradation proteins such as ribonucleases and exosome components. Two of the 17 domains are included in this category and because many general RBPs lack clear domains, additional proteins are included based upon conservation to RBPs in other organisms. Altogether, this group contains 279 genes.

### RBP-encoding genes are bound by more TFs, more RBPs, and have more splice variants

RBPs have been proposed to both fine tune gene expression as well as drive tissue and stage-specific gene expression (Blencowe 2006; Glisovic *et al.* 2008). Therefore, we hypothesized that RBPs may, as a group, be extensively regulated to mediate these functions. Here, we tested this hypothesis using the wRBP1.0 compendium and several publicly available datasets.

**Figure 2:**
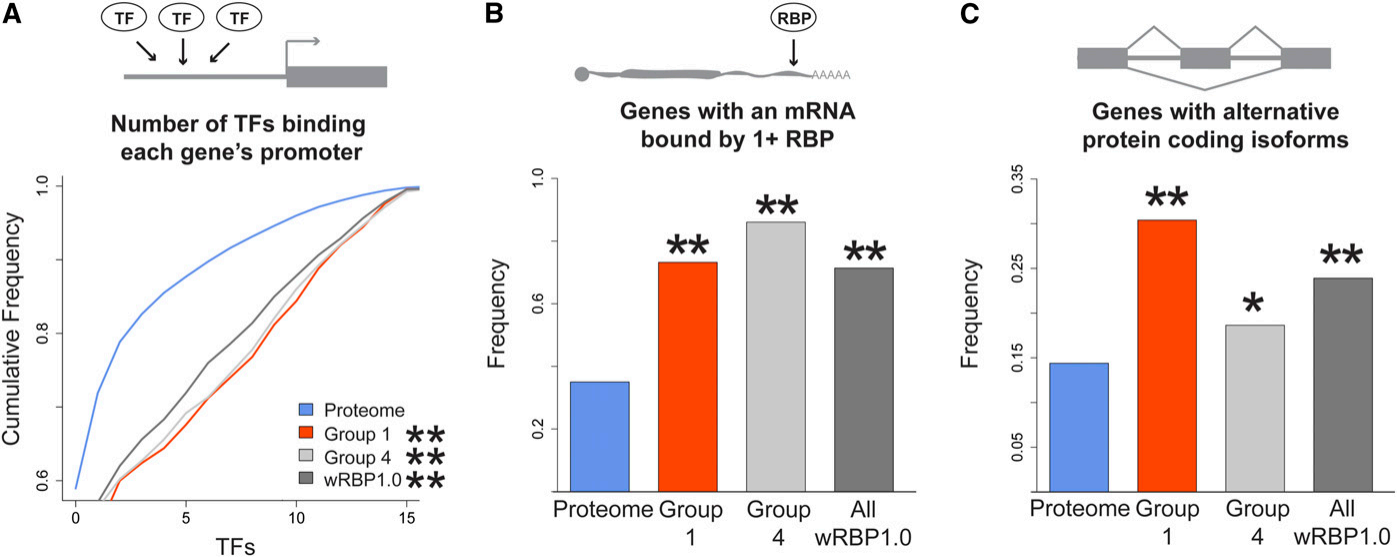
RBPs are extensively regulated by TFs and RBPs. (A) More TFs bind to RBP promoters than the promoters of other genes (B) RBPs bind to a greater proportion of RBP-encoding mRNAs. (C) RBP genes are more frequently spliced than other genes. *P < 0.05, **P < 0.005, relative to proteome, hypergeometric test (frequency data), Komologorov-Smirnov test (cumulative frequency data).

Transcriptional regulation mediated by the binding of TFs to gene promoters provides a first and important level of regulation. There are 937 predicted TFs encoded by the *C. elegans* genome (Reece-Hoyes *et al.* 2005; Reece-Hoyes *et al.* 2011), and binding of 22 of these TFs (~2%) has been examined by ChIP-seq (Gerstein *et al.* 2010). Based on these data, we found that promoters of RBP genes are bound by more TFs than promoters of other genes (Figure 2A, Figure S2). Both gene-specific and general RBP promoters are bound by significantly more TFs (*P* < 1e-9), indicating that transcriptional regulation is an important first step toward RBP expression. Importantly, these data were obtained using transgenic TF fusion strains. Because transgenes are often silenced in the germline (Cui and Han 2007) where many RBPs are expressed, it is possible that our analyses underestimate the enrichment. Further, this analysis was based on only 22 TFs; future studies will reveal the generality of our observation.

We next analyzed publicly available RBP-mRNA interactions. We obtained three RIP-Chip datasets for the *C. elegans* RBPs FBF-1, GLD-1 and RNP-8 (Kershner and Kimble 2010; Kim *et al.* 2010; Wright *et al.* 2010) and found that 73% of RBP mRNAs are bound by at least one RBP, compared with only 35% of the total transcriptome (Figure 2B). The number of RBP mRNAs from Group 4 bound is even greater (86%). Our result is consistent with Gene Ontology enrichment analysis performed in the original studies that retrieved enrichment for ‘RNA binding’ and ‘Nucleic acid binding’ terms, respectively (Kim *et al.* 2010 and Kershner and Kimble 2010).

The binding of RBPs to mRNAs affects numerous steps of an mRNA’s lifecycle, including alternative splicing (Blencowe 2006; Glisovic *et al.* 2008). To test whether *C. elegans* RBP-encoding mRNAs are more extensively spliced than other genes, we evaluated the number of protein isoforms per RBP-encoding gene by using comprehensive WormBase annotations. Approximately one-quarter of the 887 RBP-encoding genes (212; 23.9%) encode multiple isoforms, which is significantly more than the 14.4% of genes that undergo alternative splicing in the entire genome (Figure 2C). An even greater percentage of mRNAs encoding gene-specific RBPs in Group 1 are alternatively spliced (30.4%; Figure 2C). Through alternative splicing, the total number of RBPs increased by more than 40% (from 887 genes to 1242 proteins) and, interestingly, the number of distinct gene-specific RBPs increased by ~60% (250 genes encoding 401 proteins). Thus, alternative splicing increases the effective number of RBPs in the *C. elegans* proteome.

### RBP 3′ UTRs are extensively regulated

**Figure 3:**
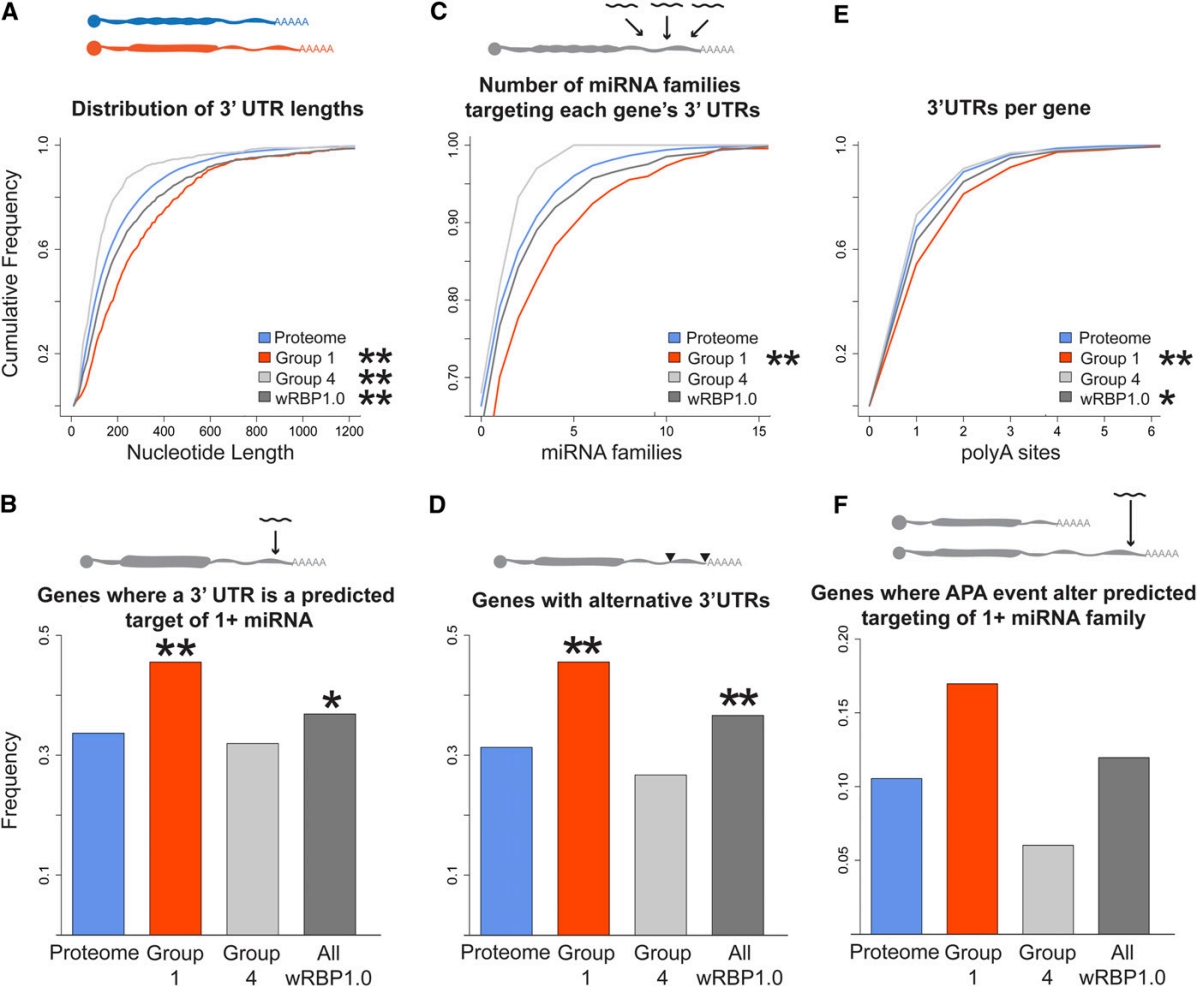
RBPs are extensively regulated through 3′ UTRs (A) RBP transcripts have longer 3′ UTRs. (B) RBP 3′ UTRs are more heavily targeted by miRNAs. (C) More miRNA families target RBP 3′ UTRs. (D) 3′ UTR annotations show that more RBPs use alternative 3′ UTRs, and (E) that RBP genes have more alternative 3′ UTRs. (F) Combined miRNA target predictions and 3′ UTR annotations reveal that APA affects predicted miRNA targeting. *P < 0.05, **P < 0.005, relative to proteome, hypergeometric test (frequency data), Komologorov-Smirnov test (cumulative frequency data).

3′ UTRs affect gene expression via interactions with RBPs and miRNAs (Bartel 2009; Kuersten and Goodwin 2003). Concordantly, *C. elegans* 3′ UTRs contain numerous conserved sequence elements that may interact with miRNAs or RBPs (Mangone *et al.* 2010; Jan *et al.* 2011). Using comprehensive 3′ UTR annotations (www.UTRome.org), we found that RBP-encoding mRNAs have significantly longer 3′ UTRs, with a median length of 156 nucleotides (nt), compared with 129 nt for the whole transcriptome (Figure 3A, Figure S2). The 3′ UTRs of gene-specific RBP mRNAs (Group 1) are even longer (215 nt), whereas general RBPs have shorter 3′ UTRs (Group 4; 100 nt). Longer 3′ UTRs can contain more regulatory sites, which implies that gene-specific RBPs may be more heavily regulated via their 3′ UTRs, whereas general RBPs may be less extensively regulated.

To test this, we first assessed the degree to which miRNAs target RBP 3′ UTRs relative to all genes. In the absence of comprehensive experimental miRNA targeting data, predictions for bound target mRNAs can be made using the miRNA seed sequences (Bartel 2009). We used target predictions from TargetScan for all *C. elegans* 3′ UTR sequences experimentally determined by 3P-Sequencing (3Pseq) (Jan *et al.* 2011). TargetScan predicts miRNA targets based upon stringent seed pairing as well as site number, type, context, and conservation (Bartel 2009). Comparison of RBP-encoding mRNA 3′ UTRs to the 3′ UTRs of all *C. elegans* mRNAs revealed that significantly more RBP 3′ UTRs are predicted targets of miRNAs (Figure 3B). Furthermore, significantly more miRNA families target each gene-specific RBP 3′ UTR compared with all 3′ UTRs, indicating a potential for increased combinatorial complexity (Figure 3C, Figure S2). In contrast, general RBPs showed no significant difference in miRNA targeting compared to the total transcriptome.

It is important to note that these predictions are based on conservation of the site in multiple species and availability of the site in folded RNA. This implies that the increased number of miRNA families targeting 3′ UTRs is not solely a consequence of 3′ UTR length. To confirm this, we compared RBP 3′ UTRs with similar length 3′ UTRs from the total transcriptome by binning 3′ UTRs by length (Figure S3). This analysis confirmed that, among the shortest 3′ UTRs (*i.e.*, the first two quartiles), more miRNAs are indeed predicted to target RBP 3′ UTRs, while we did not observe a difference for the longest 3′ UTRs.

We further evaluated miRNA targeting to RBP 3′ UTRs using predictions made by mirWIP (Hammell *et al.* 2008) and argonaute ALG-1 bound 3′ UTRs determined using cross-link immuoprecipitation (Figure S4) (Zisoulis *et al.* 2010). Both of these analyses showed that RBP 3′ UTRs are indeed more frequently targeted by miRNAs, which further supports the observations made with TargetScan predictions.

Alternative 3′ UTR usage provides additional unique sites of regulation for miRNAs and RBPs or, conversely, can eliminate regulatory sites for these same factors. Recently, it has been shown that shortening of 3′ UTRs by alternative polyadenylation (APA) alters protein expression in proliferating cells, an effect partly attributed to the loss of miRNA binding sites (Sandberg *et al.* 2008; Mayr and Bartel 2009). Using 3′ UTR annotations determined by 3P-Seq (Jan *et al.* 2011), we found that more RBPs use APA and that RBPs possess more distinct 3′ UTRs than the total transcriptome (Figures 3, D and E; results with 3′UTRome annotations were consistent, data not shown). Once again, the effect was especially pronounced for gene-specific RBPs (Group 1). We calculated the number of genes in which APA eliminates all predicted targeting sites for one or more miRNA family, thereby preventing miRNA repression and increasing gene expression. Using 3P-seq-derived 3′ UTRs and TargetScan miRNA target predictions, we found that more than 15% of the gene-specific RBPs could evade potential repression by at least one miRNA family using APA, a fraction that is more than twice that of the total transcriptome (Figure 3F). The predicted effects of APA may also affect gene expression through the distinct binding of RBPs to alternate 3′ UTRs.

### RBPs are more extensively phosphorylated

**Figure 4:**
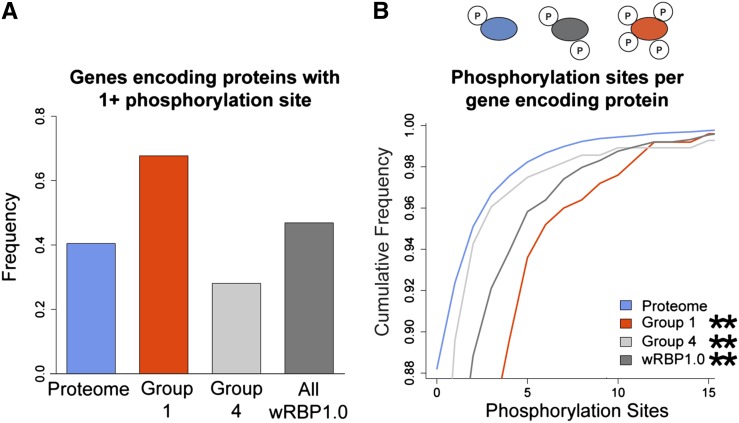
RBPs are extensively regulated posttranslationally. (A) More RBPs are phosphorylated. (B) RBPs have more phosphorylated residues per protein. *P < 0.05, **P < 0.005, relative to proteome, hypergeometric test (frequency data), Komologorov-Smirnov test (cumulative frequency data).

Posttranslational modifications provide another mechanism to create protein diversity. In particular, phosphorylation can affect the ability of proteins to function and/or interact with binding partners (Deribe *et al.* 2010). To evaluate the degree to which RBPs are phosphorylated, we interrogated phosphoproteome data that were obtained by tandem mass spectrometry of synchronized adult worms and that identified 6780 phosphorylation sites on 2373 proteins (Zielinska *et al.* 2009). Because many factors can affect the ability for certain proteins to be detected in mass spectrometry, we corrected for potential biases by normalizing the frequency of detected RBPs in each group by a separate mass spectrometry study that analyzed the proteome of mixed stage worms and did not enrich for phophopeptides (Figure S5) (Merrihew *et al.* 2008). We found that more gene-specific RBPs are phosphorylated relative to the entire proteome (Figure 4A). Furthermore, gene-specific RBPs (Group 1) have significantly more phosphorylation sites per protein than the total proteome (Figure 4B, Figure S2). In contrast, general RBPs (Group 4) are less frequently phosphorylated, although this group still contains more phosphorylation sites than entire proteome. This finding confirms the enrichment for the Gene Ontology term ‘RNA binding’ in the mass spectrometry dataset (Zielinska *et al.* 2009). The increased level of RBP phosphorylation further indicates that RBPs are indeed a heavily regulated class of cellular regulators.

### Comparison of gene-specific RBPs (Group 1) with TFs

**Figure 5:**
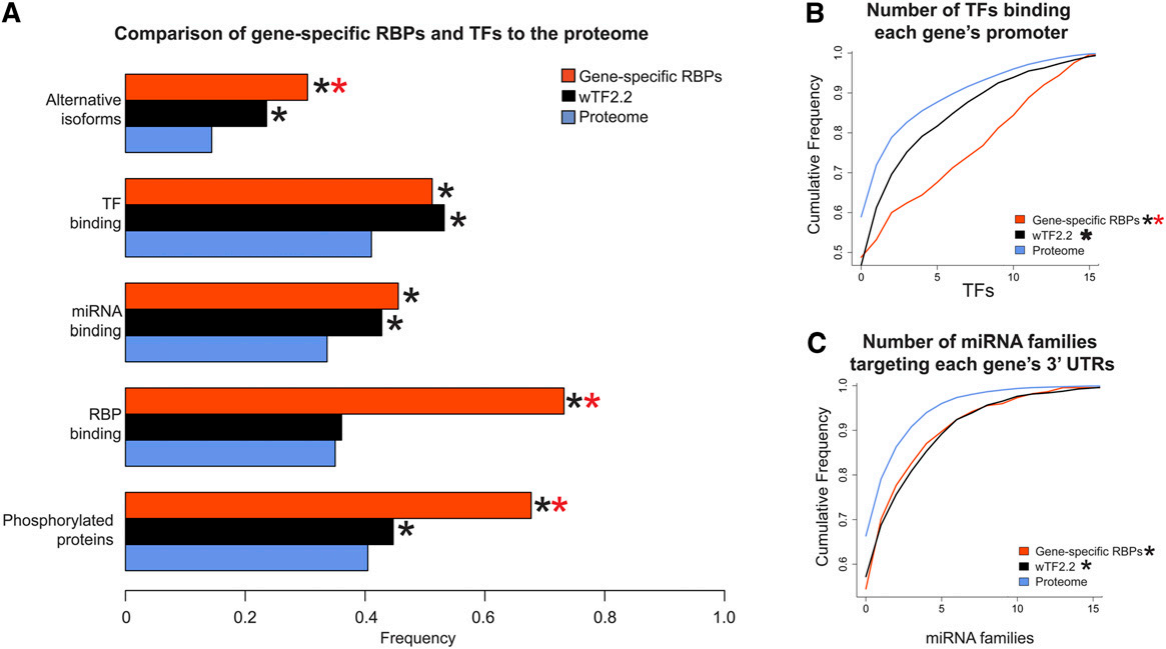
Comparison of gene-specific RBPs (Group 1) with TFs. (A) Comparison of alternative isoforms, TF binding, miRNA targeting, RBP binding, and phosphorylation. (B) Gene-specific RBPs have more TFs bound to promoters relative to TF genes. (C) Gene-specific RBPs and TFs have 3′ UTRs targeted by more miRNA families. *P < 0.005, relative to proteome, **P < 0.005 relative to wTF2.2, hypergeometric test (frequency data), Komologorov-Smirnov test (cumulative frequency data).

Group 1 RBPs are conceptually analogous to TFs in that they are predicted to bind to and regulate genes in a specific manner. Thus, we compared the characteristics of gene-specific RBPs in Group 1 to those of TFs. Although RBPs and TFs both have more isoforms than the general proteome, RBPs have significantly more isoforms compared with TFs (Figure 5A). This finding is interesting because RBPs often contain multiple RBDs that are differentially included in different isoforms, whereas most *C. elegans* TFs have only one DNA binding domain (Table S2) (Reece-Hoyes *et al.* 2005). There are more TFs bound per RBP promoter than per TF promoter, which indicates that there may be more combinatorial complexity in the transcriptional regulation of RBP genes, or in the generation of tissue-specific gene expression patterns (Figure 5B).

Both RBPs and TFs are predicted to have 3′ UTRs that are more frequently targeted by miRNAs, and there is no difference between the numbers of distinct miRNA families that target their 3′ UTRs (Figure 5, A and C). However, there was a large, significant difference involving binding by RBPs: RBP-encoding mRNAs are more frequently bound by RBPs than TF mRNAs and mRNAs in general (Figure 5A). This difference could be attributed to an expression bias since RBP mRNAs are enriched in the germline (Wang *et al.* 2009) and should therefore be re-evaluated after the determination of additional RBP-mRNA interaction data, including that of RBPs expressed in the soma. Finally, phosphorylation of both RBPs and TFs is significantly enriched relative to the proteome, but RBPs are more extensively phosphorylated than TFs. Taken together, both types of regulators are extensively regulated.

### Conclusions

We present wRBP1.0: a comprehensive compendium of *C. elegans* RBPs. As has been demonstrated for the *C. elegans* TF compendium (Reece-Hoyes *et al.* 2005, 2007; Grove *et al.* 2009; Reece-Hoyes *et al.* 2011) we expect that wRBP1.0 will be an invaluable resource for the creation of ORF-based clone collections, the delineation of RBP expression patterns, and RBP regulatory networks.

Using wRBP1.0 and several publicly available genomic, transcriptomic and proteomic datasets, we found that RBPs are extensively regulated at each level. A question that remains is why an organism extensively regulates its RBPs. One attractive possibility is that individual RBPs mediate precise gene regulation under different developmental or environmental conditions or in distinct cells or tissues within the animal. Such diverse functionalities could potentially be greatly facilitated by a combination and layering of the different transcriptional and posttranscriptional regulatory mechanisms. Furthermore, it is likely beneficial to the animal to be able to rapidly decrease the level or activity of different RBPs, such that downstream target gene expression can change rapidly as well.

Many of the regulatory trends we observed are more pronounced for gene-specific RBPs, *i.e.*, those we predict to function analogously to TFs. There are nearly four times more genes predicted to encode TFs than gene-specific RBPs in the *C. elegans* genome (937 *vs.* 251) (Reece-Hoyes *et al.* 2011; this study). Strikingly, however, gene-specific RBPs have more alternative isoforms and are more extensively phosphorylated than TFs. This finding could suggest that despite fewer gene-specific RBP genes than TF genes in the *C. elegans* genome, regulatory mechanisms can increase the repertoire of RBPs, thereby diversifying their regulatory capacity.

Related analyses have been performed in the unicellular eukaryote *Saccharomyces cerevisiae* (Mittal *et al.* 2009, 2011). Using a list of putative RBPs (Hogan *et al.* 2008), RBP mRNAs were shown to have shorter half-lives, greater abundance, and greater ribosome occupancy (Mittal *et al.* 2009). Additionally, it was shown that RBPs are more abundant, have longer half-lives, and decreased noise (Mittal *et al.* 2009). These trends were more pronounced for ribosomal RBPs and for RBPs with high connectivity, as defined by interaction data. Combined with complementary analyses in this study it is clear that RBPs exhibit properties distinct from the total transcriptome/proteome. It also is evident that gene-specific/low connectivity RBPs exhibit properties distinct from nongene-specific/high connectivity RBPs. Altogether, wRBP1.0 provides a starting point for the generation of RBP clone resources that can be used in system-level characterization of posttranscriptional regulatory networks, as well as a first step in the analysis of the regulation of this important class of proteins.

## Supplementary Material

Supporting Information
