# Supplementary material for: A Compendium of Caenorhabditis elegans RNA Binding Proteins Predicts Extensive Regulation at Multiple Levels
Source: G3 (Bethesda). 2013 Feb 1;3(2):297–304. doi: 10.1534/g3.112.004390 (PMC3564989; doi:10.1534/g3.112.004390)
Supplement: Supporting Information [file supp_3_2_297__index.html]

Supporting Information 

# A Compendium of *Caenorhabditis elegans* RNA Binding Proteins Predicts Extensive Regulation at Multiple Levels

## Supporting Information for Tamburino, Ryder, and Walhout, 2013

**Files in this Data Supplement:**

- Supporting Information - Figures S1-S5 (PDF, 427 KB)
- Figure S1 - Venn diagrams of: (A) Cross-validation of programs used by InteroProScan, and (B) initial wRBP1.0 list together with Gene Ontology and UniProtKB listed RBPs (PDF, 150 KB)
- Figure S2 - Boxplots of data shown in Figures 2 and 3, including: (A) number of TFs binding each gene's promoter, (B) distribution of 3' UTR lengths, (C) miRNA families targeting each gene's 3' UTR, and (D) number of phosphorylation sites per protein (on a gene by gene basis) (PDF, 117 KB)
- Figure S3 - Quartile binned boxplots of miRNAs targeting RBP 3' UTRs vs. 3'UTRome (PDF, 135 KB)
- Figure S4 - miRNA targeting (PDF, 82 KB)
- Figure S5 - Normalization of proteomic data (PDF, 98 KB)
- Table S1 - RBP Domains (PDF, 90 KB)
- Table S2 - wRBP1.0 Gene name, Wormbase ID, coding sequence name, domain, group, source, Gene Ontology and UniProtKB classifications (.xlsx, 60 KB)
- Table S3 - Gene metrics (.xlsx, 1 MB)
