# Supplementary material for: A Compendium of Caenorhabditis elegans RNA Binding Proteins Predicts Extensive Regulation at Multiple Levels
Source: G3 (Bethesda). 2013 Feb 1;3(2):297–304. doi: 10.1534/g3.112.004390 (PMC3564989; doi:10.1534/g3.112.004390)
Supplement: Supporting Information [file supp_3.2.297_004390SI.pdf]

G3 · Genes | Genomes | Genetics

A compendium of *C. elegans* RNA binding proteins predicts extensive regulation at multiple levels

Alex M. Tamburino[*], Sean P. Ryder[§], Albertha J.M. Walhout[*]

[*]*Program in Systems Biology and Program in Molecular Medicine,* [§]*Department of Biochemistry and Molecular Pharmacology, University of Massachusetts Medical School, Worcester, Massachusetts 01605, USA*

Corresponding author: marian.walhout@umassmed.edu

DOI: 10.1534/g3.112.004390

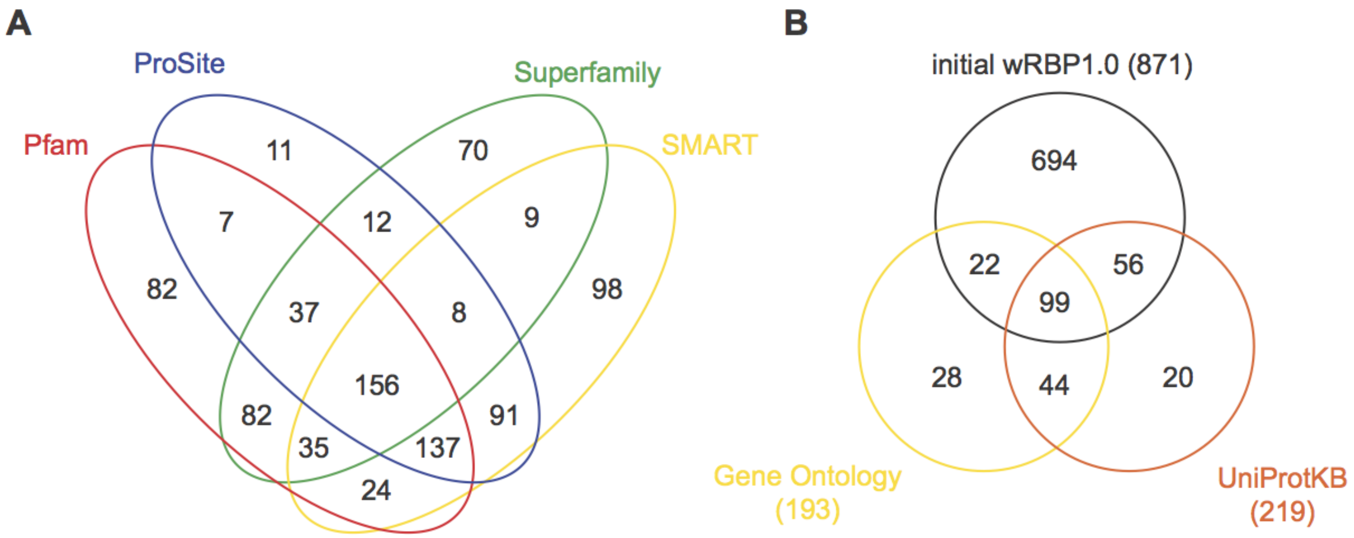

Figure S1 Venn diagrams of: (A) Cross-validation of programs used by InteroProScan, and (B) initial wRBP1.0 list together with Gene Ontology and UniProtKB listed RBPs

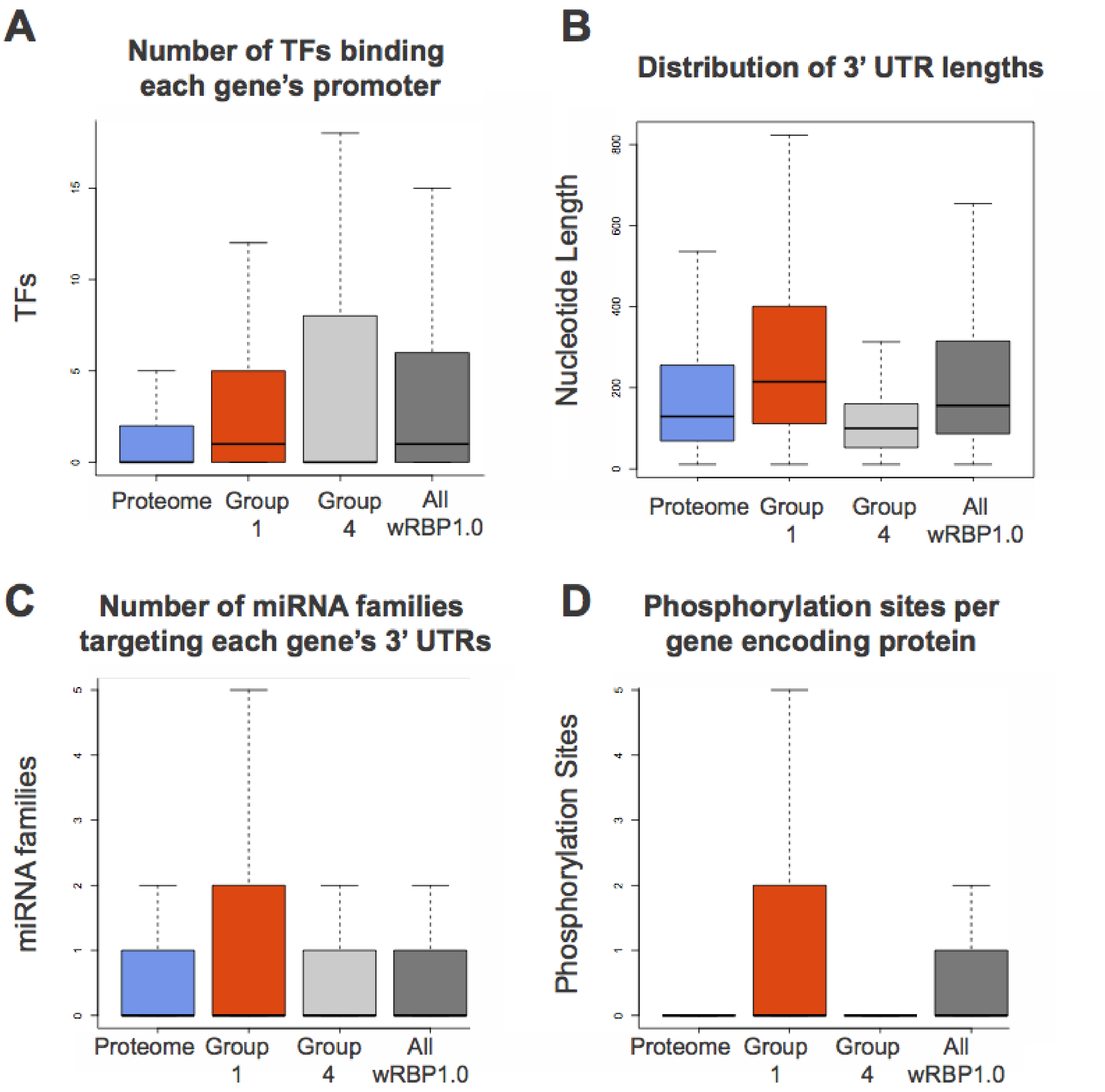

Figure S2 Boxplots of data shown in Figures 2 and 3, including: (A) number of TFs binding each gene's promoter, (B) distribution of 3' UTR lengths, (C) miRNA families targeting each gene's 3' UTR, and (D) number of phosphorylation sites per protein (on a gene by gene basis)

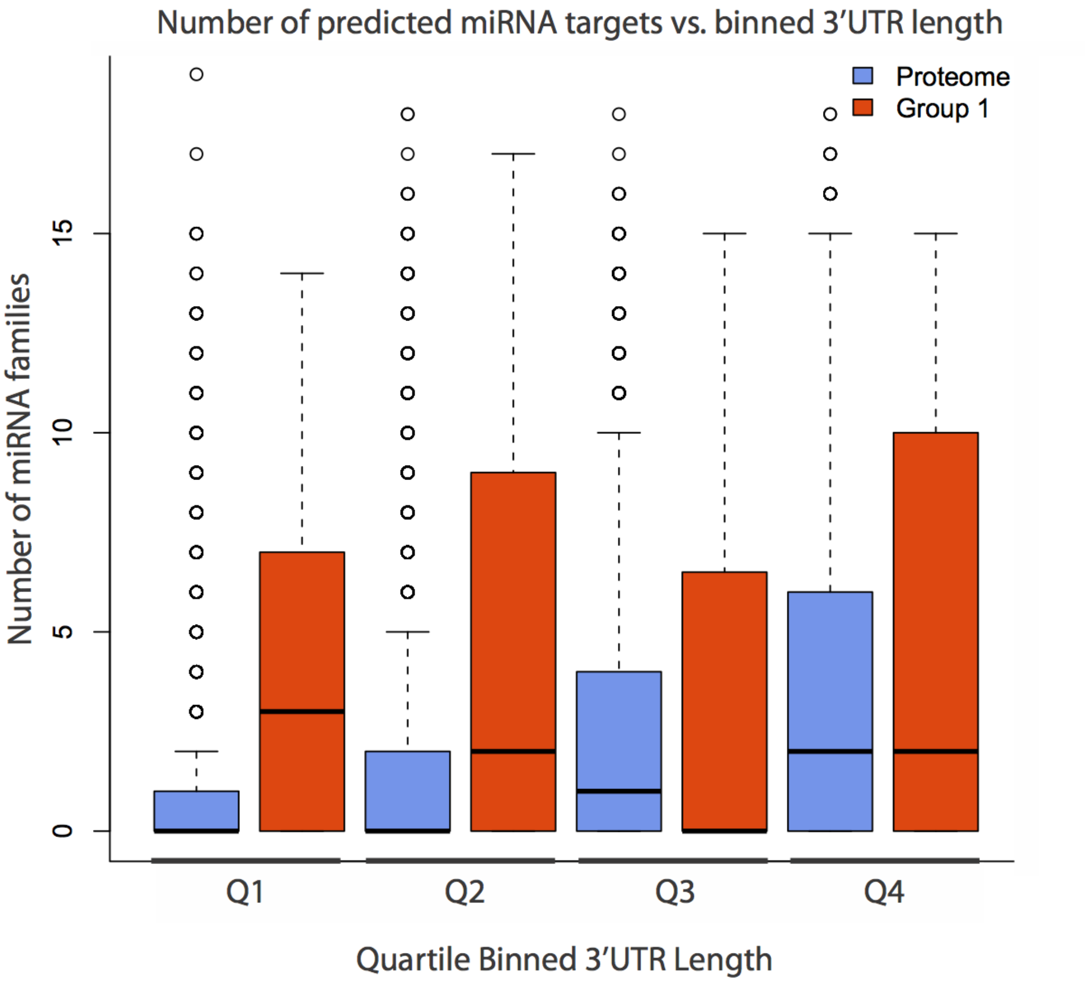

Figure S3 Quartile binned boxplots of miRNAs targeting RBP 3' UTRs *vs.* 3'UTRome. Quartiles were determined using the total 3'UTRome. The distribution of 3' UTR lengths are shown for each quartile.

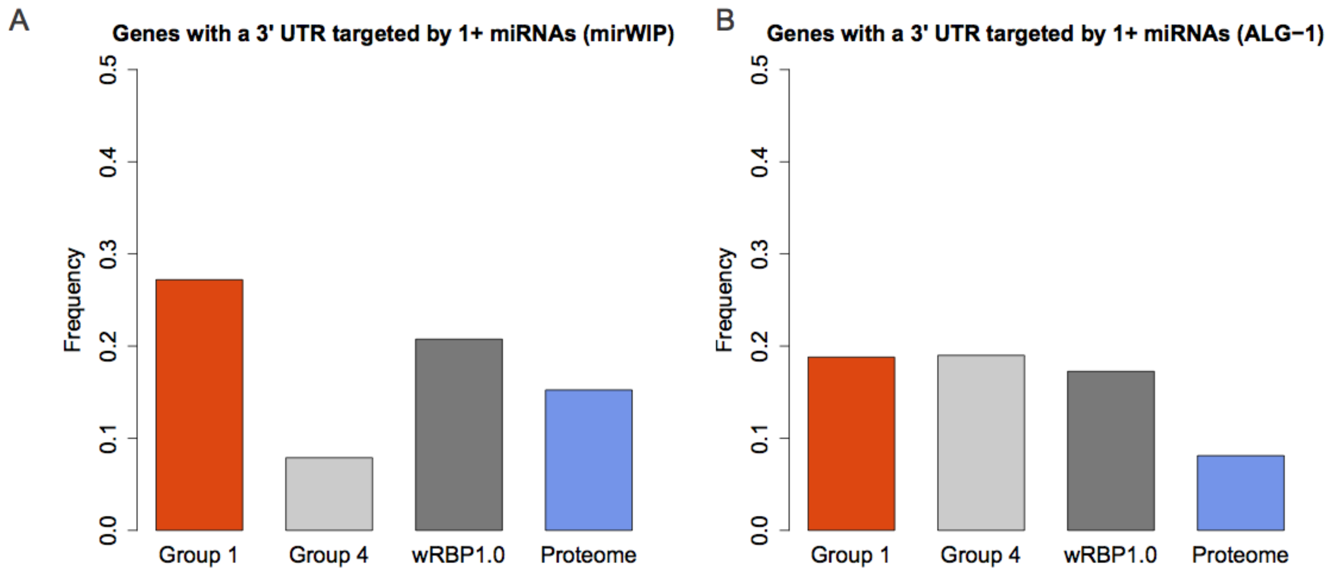

Figure S4 miRNA targeting. Frequency of 3' UTRs targeted by miRNAs according to (A) mirWIP predictions and (B) ALG-1 IP binding data

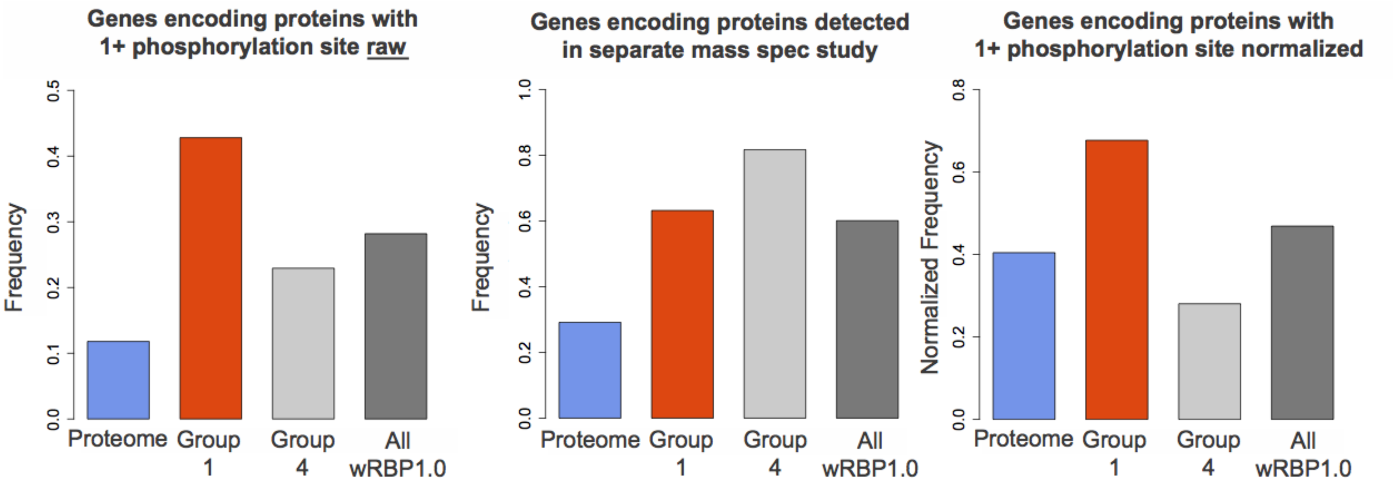

Figure S5 Normalization of proteomic data

Table S1 RBP Domains. Domain, abbreviation, group, *C.elegans* examples, example protein homologs, reference

Domain	Abbrev.	Group	*C.elegans* examples	Example protein homologs	Publications
RNA recognition motif	RRM	1	FOX-1 RNP-8	*H. sapiens* PABP, hnRNP C	Adams et al., 1986; Swanson et al., 1987; Kim et al., 2010
K homology (KH)	KH	1	MEX-3 GLD-1	*H. sapiens* hnRNP K	Siomi et al., 1993; Pagano et al., 2009; Ryder et al., 2004; Wright et al., 2010
Pumilio/FBF	PUF	1	FBF-1/2	*D. melanogaster* Pumilio	Zamore et al., 1997; Zhang et al., 1997; Bernstein et al., 2005
CCCH zinc finger	ZF_CCCH	1	POS-1 MEX-5	*H. sapiens* TTP	Carballo et al., 1998; Pagano et al., 2007; Farley et al., 2008
CCHC zinc finger	ZF_CCHC	1	LIN-28	*H. sapiens* Lin28	Balzer and Moss, 2007
Double stranded RNA binding domain	DSRBD	1	ADR-1, -2	*H. sapiens* Adar1, Adar2	Tian et al., 2004; Furic et al., 2008; Stefl et al., 2010
RGG box	RGG	1	PGL-1	*H. sapiens* hnRNP U, FMRP	Kiledjian and Dreyfuss, 1992; Ashley et al, 1993; Siomi et al., 1993
La	La	1	LARP-1 LARP-5	*H. sapiens* La	Nykamp et al., 2008; Intine et al., 2003
RNA Helicase	HEL	2	GLH-1	*D. melanogaster* VAS	Jankowsky, 2010
PAZ, PIWI, Argonautes	PAZ,PIWI	2	ALG-1 CSR-1	*D. melanogaster* PIWI	Yan et al., 2003; Kaymak et al., 2010; Cenik and Zamore, 2011
Nuclear transport factor 2	NTF2	2	NXF-2		JR Williamson (personal communication)
C2H2 zinc finger	ZF_C2H2	3	MEP-1	*X. laevis* TFIIIa	Hall, 2005; Lu et al., 2003; Belfiore et al., 2002
Sterile alpha motif	SAM	3	BCC-1	*D. melanogaster* Smaug, *S. cerevisiae* Vts1	Green et al., 2003; Aviv et al., 2003, Aviv et al., 2006
Cold shock	CSD	3	CEY-1 LIN-28	*H. sapiens* Unr	Triqueneaux et al, 1999
General factors: Translation factors, tRNA proteins, Ribosomal proteins, Ribonucleases	GF	4	IFE-4 EXO-1	*H. sapiens* eIF4G, *S. cerevisiae* Ccr4, Xrn1	Rhoads et al., 2006; Garneau et al., 2007
Sm/Lsm	Sm	4	CAR-1	*S. cerevisiae* Lsm1, Lsm2	He and Parker, 2000

Available for download at http://www.g3journal.org/lookup/suppl/doi:10.1534/g3.112.004390/-/DC1:

Table S2 wRBP1.0 Gene name, Wormbase ID, coding sequence name, domain, group, source, Gene Ontology and UniProtKB classifications.

Table S3 Gene metrics – as calculated for the entire proteome
