# Supplementary material for: A Compendium of Caenorhabditis elegans RNA Binding Proteins Predicts Extensive Regulation at Multiple Levels
Source: G3 (Bethesda). 2013 Feb 1;3(2):297–304. doi: 10.1534/g3.112.004390 (PMC3564989; doi:10.1534/g3.112.004390)
Supplement: Supporting Information [file supp_3.2.297_FigureS1.pdf]

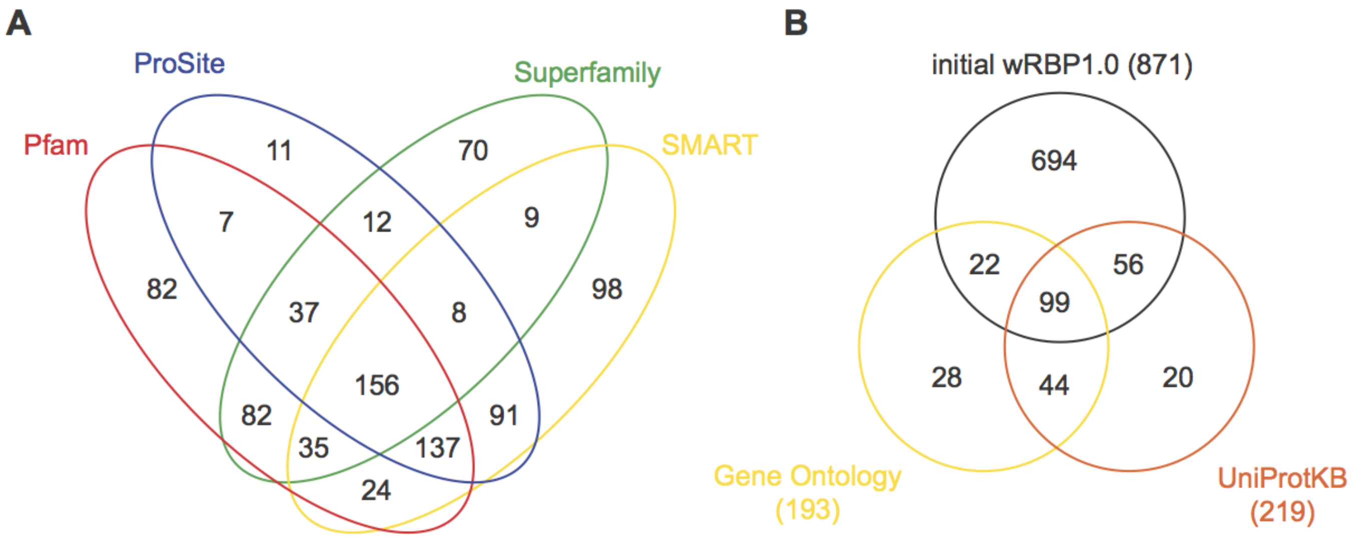

Figure S1 Venn diagrams of: (A) Cross-validation of programs used by InteroProScan, and (B) initial wRBP1.0 list together with Gene Ontology and UniProtKB listed RBPs
