# Supplementary material for: A Compendium of Caenorhabditis elegans RNA Binding Proteins Predicts Extensive Regulation at Multiple Levels
Source: G3 (Bethesda). 2013 Feb 1;3(2):297–304. doi: 10.1534/g3.112.004390 (PMC3564989; doi:10.1534/g3.112.004390)
Supplement: Supporting Information [file supp_3.2.297_FigureS2.pdf]

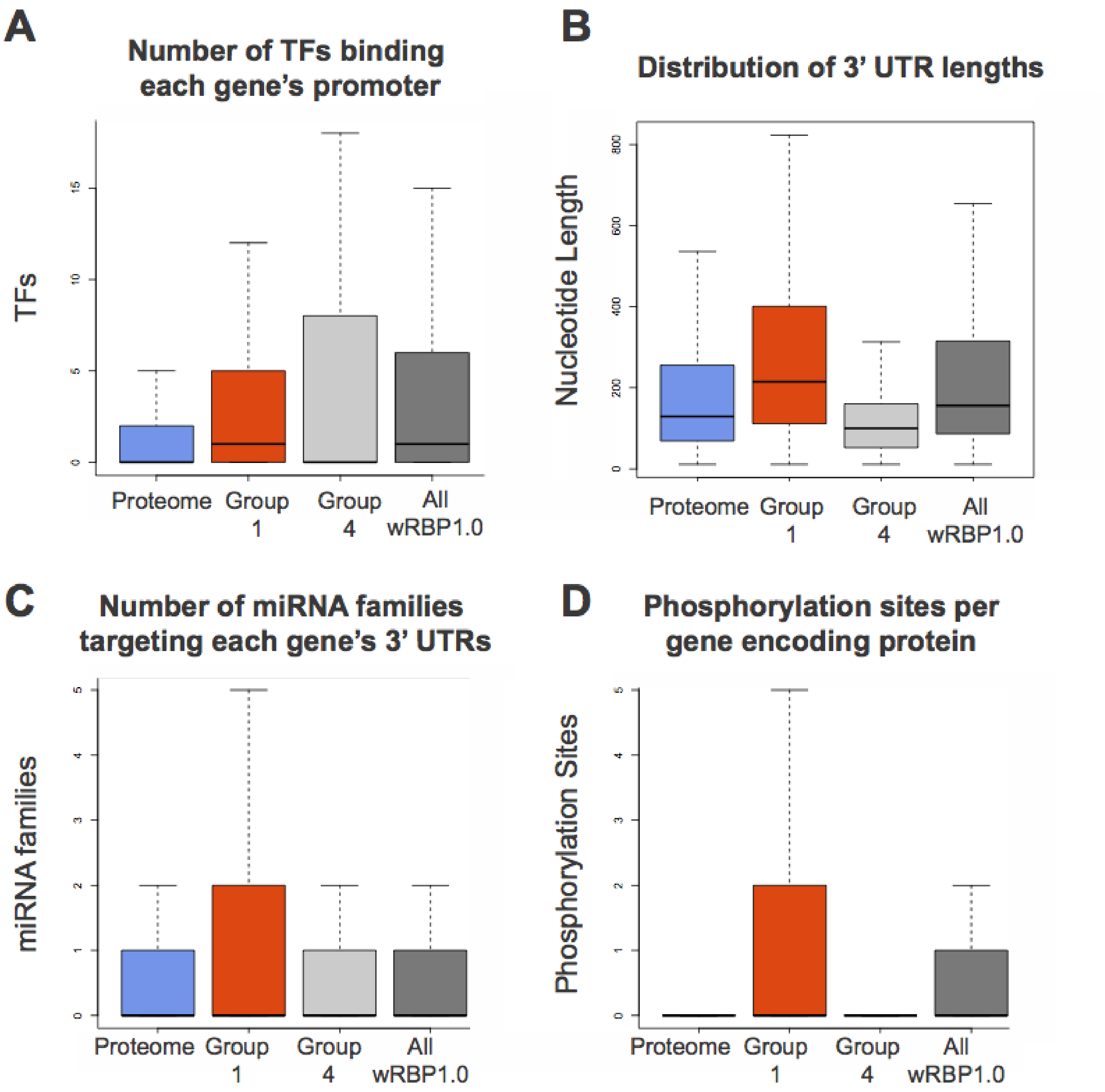

Figure S2 Boxplots of data shown in Figures 2 and 3, including: (A) number of TFs binding each gene's promoter, (B) distribution of 3' UTR lengths, (C) miRNA families targeting each gene's 3' UTR, and (D) number of phosphorylation sites per protein (on a gene by gene basis)
