# Supplementary material for: A Compendium of Caenorhabditis elegans RNA Binding Proteins Predicts Extensive Regulation at Multiple Levels
Source: G3 (Bethesda). 2013 Feb 1;3(2):297–304. doi: 10.1534/g3.112.004390 (PMC3564989; doi:10.1534/g3.112.004390)
Supplement: Supporting Information [file supp_3.2.297_FigureS3.pdf]

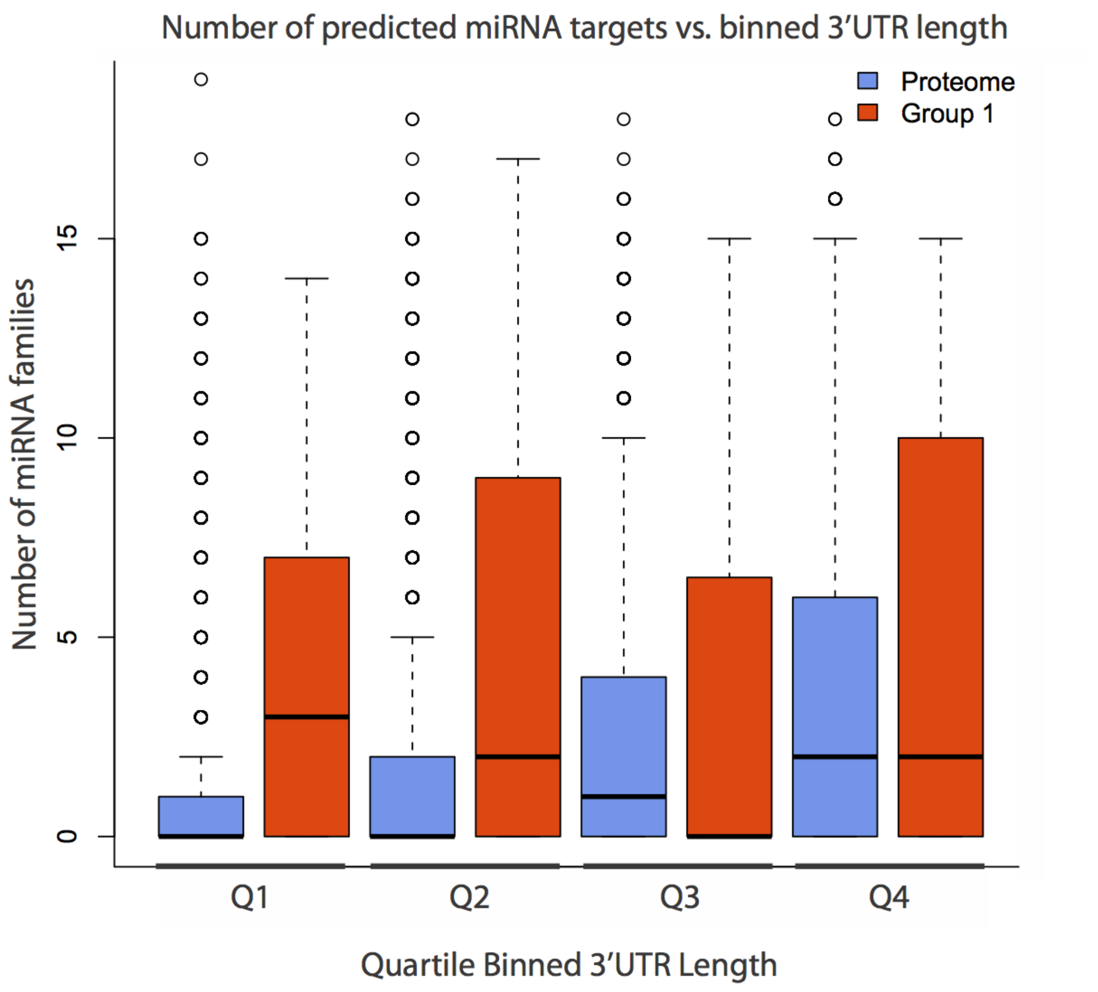

Figure S3 Quartile binned boxplots of miRNAs targeting RBP 3' UTRs *vs.* 3'UTRome. Quartiles were determined using the total 3'UTRome. The distribution of 3' UTR lengths are shown for each quartile.
