# Supplementary material for: A Compendium of Caenorhabditis elegans RNA Binding Proteins Predicts Extensive Regulation at Multiple Levels
Source: G3 (Bethesda). 2013 Feb 1;3(2):297–304. doi: 10.1534/g3.112.004390 (PMC3564989; doi:10.1534/g3.112.004390)
Supplement: Supporting Information [file supp_3.2.297_FigureS4.pdf]

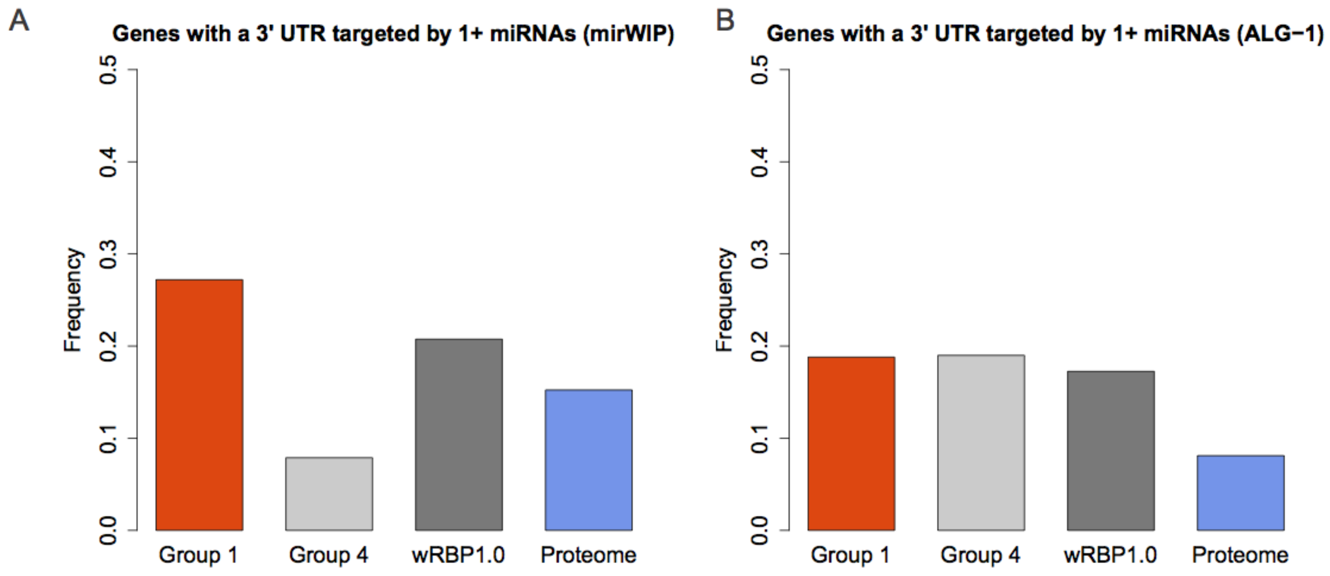

Figure S4 miRNA targeting. Frequency of 3’ UTRs targeted by miRNAs according to (A) mirWIP predictions and (B) ALG-1 IP binding data
