# Supplementary figures and images for: A Compendium of Caenorhabditis elegans RNA Binding Proteins Predicts Extensive Regulation at Multiple Levels
Source: G3 (Bethesda). 2013 Feb 1;3(2):297–304. doi: 10.1534/g3.112.004390 (PMC3564989; doi:10.1534/g3.112.004390)

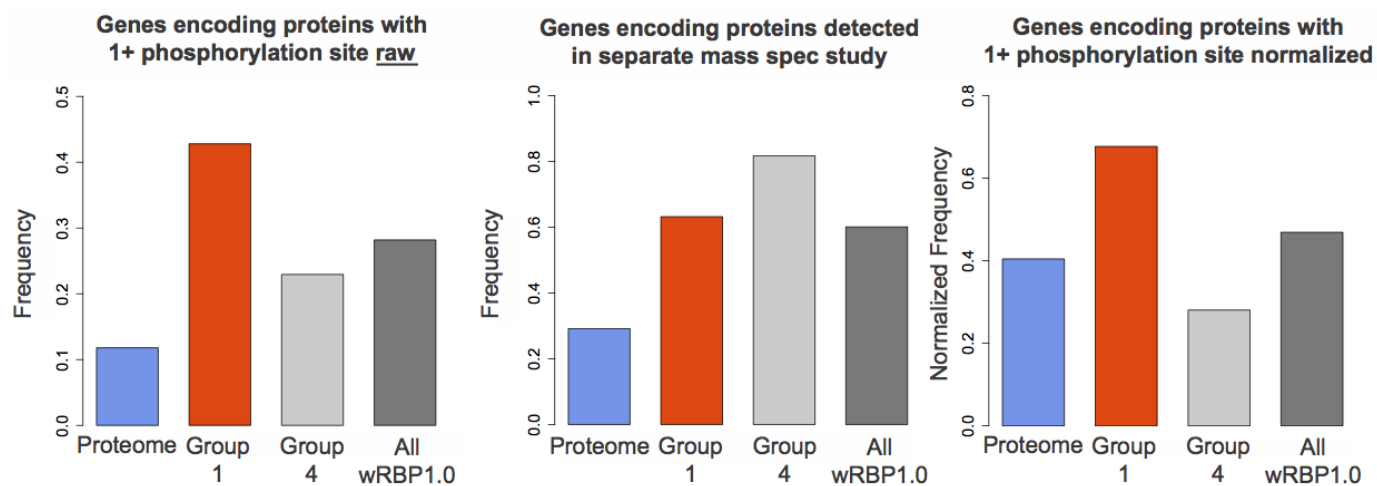

**Figure S5** Normalization of proteomic data

Supplement: Supporting Information [file supp_3.2.297_FigureS5.pdf]
