# Supplementary material for: A Compendium of Caenorhabditis elegans RNA Binding Proteins Predicts Extensive Regulation at Multiple Levels
Source: G3 (Bethesda). 2013 Feb 1;3(2):297–304. doi: 10.1534/g3.112.004390 (PMC3564989; doi:10.1534/g3.112.004390)
Supplement: Supporting Information [file supp_3.2.297_TableS1.pdf]

Table S1 RBP Domains. Domain, abbreviation, group, *C.elegans* examples, example protein homologs, reference

Domain	Abbrev.	Group	*C.elegans* examples	Example protein homologs	Publications
RNA recognition motif	RRM	1	FOX-1 RNP-8	*H. sapiens* PABP, hnRNP C	Adams et al., 1986; Swanson et al., 1987; Kim et al., 2010
K homology (KH)	KH	1	MEX-3 GLD-1	*H. sapiens* hnRNP K	Siomi et al., 1993; Pagano et al., 2009; Ryder et al., 2004; Wright et al., 2010
Pumilio/FBF	PUF	1	FBF-1/2	*D. melanogaster* Pumilio	Zamore et al., 1997; Zhang et al., 1997; Bernstein et al., 2005
CCCH zinc finger	ZF_CCCH	1	POS-1 MEX-5	*H. sapiens* TTP	Carballo et al., 1998; Pagano et al., 2007; Farley et al., 2008
CCHC zinc finger	ZF_CCHC	1	LIN-28	*H. sapiens* Lin28	Balzer and Moss, 2007
Double stranded RNA binding domain	DSRBD	1	ADR-1, -2	*H. sapiens* Adar1, Adar2	Tian et al., 2004; Furic et al., 2008; Stefl et al., 2010
RGG box	RGG	1	PGL-1	*H. sapiens* hnRNP U, FMRP	Kiledjian and Dreyfuss, 1992; Ashley et al, 1993; Siomi et al., 1993
La	La	1	LARP-1 LARP-5	*H. sapiens* La	Nykamp et al., 2008; Intine et al., 2003
RNA Helicase	HEL	2	GLH-1	*D. melanogaster* VAS	Jankowsky, 2010
PAZ, PIWI, Argonautes	PAZ,PIWI	2	ALG-1 CSR-1	*D. melanogaster* PIWI	Yan et al., 2003; Kaymak et al., 2010; Cenik and Zamore, 2011
Nuclear transport factor 2	NTF2	2	NXF-2		JR Williamson (personal communication)
C2H2 zinc finger	ZF_C2H2	3	MEP-1	*X. laevis* TFIIIa	Hall, 2005; Lu et al., 2003; Belfiore et al., 2002
Sterile alpha motif	SAM	3	BCC-1	*D. melanogaster* Smaug, *S. cerevisiae* Vts1	Green et al., 2003; Aviv et al., 2003, Aviv et al., 2006
Cold shock	CSD	3	CEY-1 LIN-28	*H. sapiens* Unr	Triqueneaux et al, 1999
General factors: Translation factors, tRNA proteins, Ribosomal proteins, Ribonucleases	GF	4	IFE-4 EXO-1	*H. sapiens* eIF4G, *S. cerevisiae* Ccr4, Xrn1	Rhoads et al., 2006; Garneau et al., 2007
Sm/Lsm	Sm	4	CAR-1	*S. cerevisiae* Lsm1, Lsm2	He and Parker, 2000
